# Early life parameters and personality affect oxidative status during adulthood in an altricial rodent

**DOI:** 10.14814/phy2.15427

**Published:** 2022-10-05

**Authors:** Heiko G. Rödel, Veridiana Jardim, Marylin Rangassamy, Ludivine Jaravel, Daphné Jacquet, Raquel Monclús, Christophe Féron, David Costantini

**Affiliations:** ^1^ Laboratoire d'Ethologie Expérimentale et Comparée UR 4443 (LEEC) Université Sorbonne Paris Nord Villetaneuse France; ^2^ Laboratory of Ethology, Ecology and Evolution of Social Insects, Department of Experimental Psychology University of Sao Paulo São Paulo Brazil; ^3^ Unité Physiologie Moléculaire et Adaptation (PhyMA) Muséum National d'Histoire Naturelle, CNRS, CP32 Paris France

**Keywords:** DNA damage, exploration tendency, litter size, mound‐building mouse, *Mus spicilegus*, oxidative stress

## Abstract

It is increasingly recognized that alterations of the cellular oxidative status might be an important cost underlying challenging early life conditions. For example, an increased litter size can impose challenges as the offspring will face increased competition for maternal resources. Within a litter, individuals with relatively higher starting mass typically show higher growth rates, which can lead to increased oxidative damage. We investigated the long‐term consequences of these early life parameters on the oxidative status in mature mound‐building mice (*Mus spicilegus*). Individual differences in the animals' exploration tendency were assessed by repeated open field and novel object tests. We predicted less exploratory phenotypes, which typically show a higher stress responsiveness, to be particularly susceptible to possible effects of these early life parameters on oxidative status. We quantified oxidative damage of DNA (8‐hydroxy‐2'‐deoxyguanosine levels, 8‐OHdG) and proteins (protein carbonyl content, PCC), and activities of the antioxidants catalase (CAT), glutathione peroxidase (GPx), and superoxide dismutase (SOD) in liver and skeletal muscle tissue. 8‐OHdG levels were positively associated with CAT and SOD in both tissues, indicating that increased oxidative DNA damage was associated with an upregulation of antioxidant production. Hepatic DNA damage after maturity was increased in animals from larger litters. In less exploratory animals, DNA damage and the activity of CAT and SOD in the muscle were increased, but only in individuals with higher relative starting mass (measured on postnatal day 9). This interaction may be explained by the typically higher adrenocortical activity in less exploratory phenotypes and by the higher growth in relatively heavier pups, two factors known to increase oxidative stress. These findings contribute to enlightening the complex interplay between early life conditions, personality, and oxidative status.

## Graphical abstract

Hepatic DNA damage was increased in adult mound‐building mice (*Mus spicilegus*) from larger litters, possibly due to increased sibling competition in such animals during early life. In less exploratory animals, DNA damage in skeletal muscle tissue was increased, but only in individuals with higher pup body mass relative to litter siblings, possibly due to the higher adrenocortical activity in less exploratory phenotypes and the higher growth in heavier pups, two factors known to increase oxidative stress. DNA damage levels were positively associated with catalase and superoxide dismutase activities in liver and muscle tissue, indicating that increased oxidative damage was associated with an upregulation of antioxidant production.

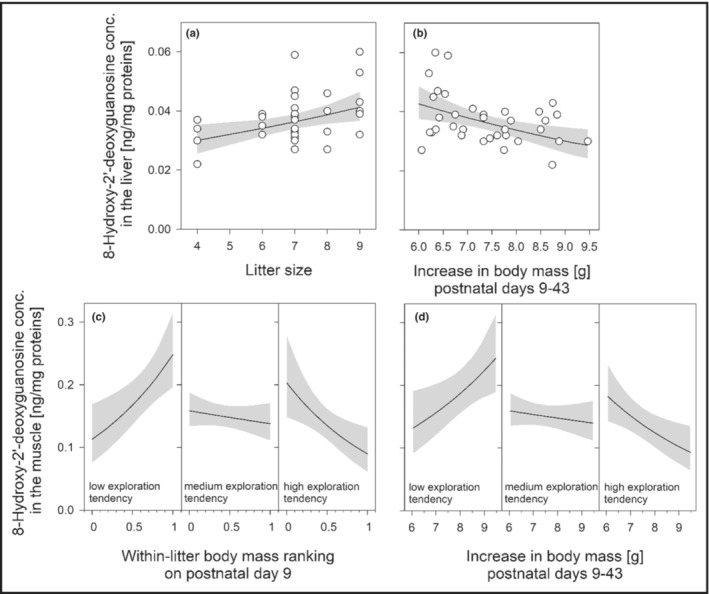

## 1 INTRODUCTION

Challenging conditions experienced during early development can have profound, and even life‐long consequences on an individual's health, reproduction, and survival (Costantini & Marasco, 2022; Lindström, 1999; Lummaa & Clutton‐Brock, 2002; Metcalfe & Monaghan, 2001; Mousseau & Fox, 1998). It has been suggested that one of the key mechanisms involved in the mediation of such long‐term effects might be oxidative stress, that is, an increased production of reactive oxygen species (ROS) relative to the body's antioxidant defense (Costantini, 2008, 2019; Romero‐Haro & Alonso‐Alvarez, 2020; Selman et al., 2012; Smith et al., 2016; Yang et al., 2020). Such an imbalance of the redox system, resulting in oxidative damage in different cells and tissues can accelerate aging and limit reproductive success, and can contribute to the development of various chronic diseases (de Araújo et al., 2016; Liguori et al., 2018; Romano et al., 2010; Yousefzadeh et al., 2021).

A first essential step to better understanding the possible mediating role of oxidative stress in life‐history trade‐offs and phenotypic programming is to explore long(er)‐term consequences of early life conditions on parameters of oxidative status (Song et al., 2009). In this context, litter or brood size have been highlighted as a prominent factor shaping the early developmental environment in mammals and birds. Competition among offspring for restricted maternal/parental food resources is usually higher in larger litters or broods (Mock & Parker, 1997). In altricial mammals, this is evident by the typically lower growth rate in offspring from larger litters at least until weaning, as the share of milk obtained by an individual pup typically decreases with increasing litter size (Koskela, 1998; Machin & Page, 1973; Mendl, 1988; Rödel, Prager, et al., 2008).

The stress‐inducing character of increased sibling competition in enlarged broods or larger litters is further supported by studies reporting higher glucocorticoid levels prior to weaning or fledging in offspring developing under such conditions (birds: Gil et al., 2019; Hardt et al., 2017; Vitousek et al., 2017; mammals: Cohas et al., 2021; Fey & Trillmich, 2008, but see: Kozlowski & Ricklefs, 2011; Rödel et al., 2010). Accordingly, as chronic stress is known to deteriorate an animal's oxidative status (Costantini et al., 2011), several studies have found indications of higher oxidative damage and/or lower antioxidant activity in offspring from larger or enlarged broods or litters (Bourgeon et al., 2011; Costantini et al., 2006; Gibson et al., 2015; Gil et al., 2019, but see: López‐Arrabé et al., 2016; Losdat et al., 2010).

The relative differences in starting mass among litter siblings (i.e., within a litter) can be another relevant feature of the early development potentially affecting an individual's oxidative status. Such within‐litter differences can lead to a cascade of effects reinforcing differences in growth between siblings (Hudson et al., 2011). In general, the increased metabolic activity during periods of increased growth comes at some expenses, as it can lead to increased ROS production, thus causing oxidative damage to cells and tissue (Alonso‐Alvarez et al., 2007; Smith et al., 2016). Studies in some species of altricial small mammals have shown that pups with a relatively higher body mass at birth get access to a higher share of milk, and also consistently occupy central positions in the litter huddle, while relatively smaller ones are more often displaced to the periphery (European rabbit *Oryctolagus cuniculus*: Bautista et al., 2015, laboratory rat *Rattus norvegicus*: Bautista et al., 2010, house mouse *M. musculus*: Zepeda et al., 2018). As a consequence, relatively heavier offspring profit more frequently from positions in the warmer and energetically more favorable center of the huddle, resulting in a higher conversion of mother's milk into biomass, thus further contributing to their faster early growth (Rödel, Bautista, et al., 2008; Zepeda et al., 2019).

Such early differences in body mass among siblings can even persist into later life stages, possibly due to different growth trajectories between heavier and lighter siblings (e.g., European rabbits: Rödel et al., 2020). However, there are examples that individuals experiencing growth‐restricting conditions early in life might accelerate their growth later on, to at least partly catch up in body mass and size (Finkielstain et al., 2013; Metcalfe & Monaghan, 2001). Thus, when studying the potential consequences of increased early growth rates on parameters of oxidative status, it may be useful to consider potential periods of accelerated catch‐up growth during postweaning life.

The individual behavioral phenotype is a further important aspect which may alter or modulate an animal's physiological response to a challenge. Studies in mammals and birds have shown a generally higher or more chronic activation of the hypothalamic–pituitary–adrenal (HPA) axis in more reactive, less aggressive and less exploratory phenotypes, as opposed to more proactive (Koolhaas et al., 1999), aggressive (Veenema et al., 2003) and exploratory ones (Baugh et al., 2017; Carere et al., 2003; Lavergne et al., 2019; Lendvai et al., 2011; Montiglio et al., 2012; Rossi et al., 2018; Stöwe et al., 2010). An increased HPA axis activity, releasing higher levels of circulating glucocorticoids, is well known for its potential to induce cellular oxidative stress (Costantini et al., 2011; Spiers et al., 2015). The typically higher resting metabolic rate of more reactive and less exploratory animals (Hürlimann et al., 2019; Réale et al., 2010) may further contribute to a higher ROS production in such individuals (Frisard & Ravussin, 2006). This might be one of the main mechanisms leading to associations between certain personality traits and oxidative profiles, as it has been shown in several studies on vertebrates, including humans (Matsuzawa et al., 2005; Vida et al., 2018). For example, studies in non‐human vertebrates indicate a tendency that more reactive, less aggressive, and less exploratory individuals have a lower antioxidant capacity compared to more proactive, aggressive, and exploratory ones (Costantini et al., 2012; Herborn et al., 2011; Isaksson et al., 2011, but see: Costantini et al., 2008).

Thus, considering aspects of an individual's behavioral phenotype could significantly add to our understanding of possible downstream effects of early life challenges on its oxidative status during later life. We carried out such a multifactorial approach for the first time, investigating long‐term effects of litter size and of within‐litter differences in starting body mass as proxies of the conditions experienced during early life, and of exploratory tendency as a key behavioral (“personality”) trait commonly used to phenotype mammals and birds (Carere & Maestripieri, 2013; Réale et al., 2007; Rödel et al., 2015). We used the mound‐building mouse (*Mus spicilegus*) as study species, an altricial and polytocous small rodent showing clear and consistent individual differences in its tendency to explore novel environments and objects (Duparcq et al., 2019; Jardim et al., 2021). Mound‐building mice give birth to litters of variable sizes (4–11 pups, Sokolov et al., 1998), and in our study, we made use of the natural variation in this parameter; thus, we did not manipulate litter size. For evaluating the animals' oxidative status, we quantified (a) from liver and skeletal muscle tissues two parameters of oxidative damage, 8‐hydroxy‐2'‐deoxyguanosine [8‐OHdG] mutagenic DNA damage (Takeuchi et al., 1996) and protein carbonyl content [PCC] as a marker of protein damage (Dalle‐Donne et al., 2003). Damage caused by ROS has important implications in various tissues, although DNA and proteins in the liver have been reported to be among the cellular structures primarily affected by oxidative damage (Cichoż‐Lach & Michalak, 2014). Furthermore, we chose to sample skeletal muscle tissue due to the high functional importance to maintain the body's muscle integrity (Marasco et al., 2021). In these tissues, we also measured (b) three markers of enzymatic antioxidant activity reflecting the organism's performance to remove harmful ROS (Ighodaro & Akinloye, 2018), superoxide dismutase [SOD], catalase [CAT], and glutathione peroxidase [GPx].

Our main goal was to investigate whether individual traits as well as conditions experienced during early life exert long‐term consequences on the oxidative status, thus evident during adulthood. Accordingly, we (*i*) expected indications of higher oxidative stress, that is, higher oxidative damage and/or lower antioxidant activities, in animals from larger litters. We (*ii*) explored the effects of within‐litter differences in starting mass (measured on postnatal day 9) on parameters of oxidative status. Since an accelerated growth can entail oxidative costs (Smith et al., 2016), we expected faster‐growing individuals to show higher levels of oxidative damage and/or lower activities of antioxidants. On the one hand, higher growth rates until around weaning (Rödel, Prager, et al., 2008; Zepeda et al., 2019) and possibly beyond can be expected in individuals with a relatively higher starting body mass, with potential negative consequences on their oxidative status. On the other hand, animals with lower starting body mass may show increased catch‐up growth after weaning, possibly leading to increased oxidative costs. Furthermore, we (*iii*) predicted that negative consequences of early life conditions may be more pronounced in behavioral phenotypes well known for their comparatively higher HPA axis activity, that is, in less exploratory individuals compared to more exploratory ones (Baugh et al., 2017; Lavergne et al., 2019; Montiglio et al., 2012). To this end, animals were behaviorally phenotyped for individual differences in exploration tendency by repeated open field and novel object tests (Duparcq et al., 2019; Jardim et al., 2021).

## 2 METHODS

### 2.1 Study animals

We studied mound‐building mice of wild origin, descendants from animals caught in Hungary in 1999 and bred at the animal facilities of the Laboratoire d'Ethologie Expérimentale et Comparée at the Université Sorbonne Paris Nord, France. Every 4–5 years since then, additional animals have been captured in the same region in Hungary and integrated into the breeding stock to maintain genetic variation (details in [Duparcq et al., 2019]).

Animals were kept in polycarbonate cages (32.5 × 16.5 cm and 14.2 cm high, Plexx, Elst, The Netherlands) with a layer of wood shavings, in a room with a temperature of around 20 ± 1°C and a 14/10 light/dark cycle (red light on at 12:30 pm). Cages were enriched with two cardboard rolls (length: 10 cm, diameter: 6 cm) and three cotton balls per individual as material for building the nest. Food (rodent standard diet; Special Diet Services type M20, Witham, Essex, UK) and water were provided ad libitum.

We used *n* = 35 females originating from 15 litters (from 15 different mothers), with litter sizes between four and nine individuals and 1–5 females per litter (more details in the *Ethics note*). On postnatal day 9, animals were individually marked with different symbols drawn on their backs, using a black permanent non‐toxic hair dye (Prodye, Weaver Leather Livestock, Ohio, USA; see Rangassamy et al., 2015 for details on this procedure). These symbols were checked every few days and were redrawn if necessary to keep individual identities. Furthermore, animals were weighed individually on that day (postnatal day 9), as well as on postnatal days 32 (first behavioral test session), 43 (second behavioral test session), and when they were killed off (around postnatal day 145). On postnatal day 28, animals were weaned, and their sex was determined by external genital inspection. After that, they were kept in mixed‐sex sibling groups until day 55, shortly before this species reaches maturity (Busquet et al., 2009). Afterward, females were kept in same‐sex groups of two to three individuals (usually litter sisters) per cage (*n* = 17 cages). Two litters of more than four sisters were split up into different cages, and four single females were merged together into two cages. Thus, for statistical analysis, we used the cage identity (next to litter identity) as a random intercept factor to consider possible differences among the different cages (see below).

### 2.2 Behavioral phenotyping

Individual differences in exploratory activity were assessed by repeated behavioral tests, by the combination of an open field test and directly after by a novel object test. Both tests were carried out two times, on postnatal day 32 (*T*_1_) and on day 43 (*T*_2_). The apparatus was made of white polyethylene and was used for both tests. It consisted of a circular open field arena with a diameter of 60 cm surrounded by walls with a height of 65 cm. All behavioral tests were carried out during the dark (red light) phase, that is, during the activity period of this species. More details on procedures are given in (Jardim et al., 2021). The behavioral tests were carried out with *N* = 35 focal females from which oxidative status parameters were taken.

#### 2.2.1 Open field test

During each of the two test repeats, individuals were placed singly in a defined peripheral position at the edge of the arena and were video recorded for 5 min while exploring the arena. From the video footage, we quantified the total distance covered using the software Ethovision, version XT10 (Noldus Information Technology, Wageningen, The Netherlands).

#### 2.2.2 Novel object test

After 5 min of open field test, the individual was caught and kept in an opaque plastic box inside the arena for around 20 s, while a novel object was introduced into the center of the test arena. The individual was then released from the box into the arena. After 5 min of testing, animals were caught and returned to their home cage. On postnatal day 32 the mice were confronted with a small transparent and round glass pot, and on postnatal day 43 with a rounded kidney‐shaped metallic box (details in Jardim et al., 2021). Video footage was analyzed using the software Boris, version 7.9.8 (Friard & Gamba, 2016). We recorded (a) the latency to touch the novel object and (b) the time subjects spent exploring the object by climbing and moving while being on top of it.

### 2.3 Measurements of parameters of oxidative status

Around postnatal day 145 (±2 days), the animals were killed off by decapitation. Immediately after, animals were dissected and the muscles of both hindlegs and the liver were taken. These tissue samples were stored in 2‐ml‐labeled plastic tubes and were immediately frozen and stored at −80°C.

Three to four months later, samples were defrosted. We homogenized liver and muscle samples, separately, in Dulbecco's Phosphate‐Buffered Saline (Sigma‐Aldrich, France) supplemented with 1 mM of phenylmethylsulfonyl fluoride (Sigma‐Aldrich, France) as an inhibitor of proteases using a TissueLyser II (Qiagen) at 30 Hz for 1 min. Afterward, we centrifuged tubes for 10 min at 4°C to obtain clean supernatants to be used for the assays. We measured (*i*) the concentration of 8‐hydroxy‐2'‐deoxyguanosine (8‐OHdG; marker of oxidative DNA damage with mutagenic properties) using the 8‐hydroxy‐2'‐deoxyguanosine ELISA Kit (Abcam, France), (*ii*) the concentration of protein carbonyls [PCC] using the Protein Carbonyl Content Assay Kit (Abcam, France), (*iii*) the activity of the antioxidant enzyme catalase [CAT] using the Catalase Activity Assay Kit (Abcam, France), (*iv*) the activity of the antioxidant enzyme glutathione peroxidase [GPx] using the Ransel assay (Randox Laboratories, France) and (*v*) the activity of the antioxidant enzyme superoxide dismutase [SOD] using the Ransod assay (Randox Laboratories, France). We standardized values of markers by the concentration of proteins as quantified using the Bradford protein assay with albumin as reference standard (Sigma‐Aldrich, France). All assays were run according to manufacturer's instructions. Concentrations of markers were expressed as: ng/mg proteins for 8‐OHdG, nmol/mg proteins for PCC, nmol H_2_O_2_/min/mg proteins for CAT, and units/mg proteins for GPx and SOD.

### 2.4 Ethics note

Animals were kept and treated according to accepted international standards (Vitale et al., 2018) and to the ethics and animal care guidelines of France, where the experiments were carried out. Experimental procedures were approved by the French Ethics Committee for Animal Experimentation “Charles Darwin” (APAFIS#17922–2,018,112,916,198,301 v8) and by the ethics committee of our institution (SBEA‐LEEC‐USPN). Thirty‐five females were bred especially for this study and the remaining male siblings were used for another research project (Jardim et al., 2021). At around postnatal day 145, these females were killed off by decapitation to obtain tissue samples for the analysis of parameters of oxidative status.

### 2.5 Statistical analysis and sample sizes

Statistical analyses were carried out with the program R, version 4.0.3 (R Core Team, 2021). All statistical tests reported in this study are two‐tailed. The statistical units were the values obtained from the different study animals, details on sample sizes below.

We tested for litter size effects on (a) pup body mass on postnatal day 1 (*n* = 87 litters with a total of 662 pups, from 51 breeding pairs) and (b) the increase in body mass from postnatal day 1 to day 20 (*n* = 34 litters with a total of 263 pups, from 28 breeding pairs), using a larger dataset from our mound‐building mouse breeding facility database. For these analyses, averaged values over all pups per litter were used. We fitted linear mixed‐effects models (LMM) using the R package *lme4* (Bates et al., 2015) including the identity of the parental pair as a random intercept factor. Response variables were right skewed, resulting in non‐normally distributed model residuals. Thus, data were log[*x*] transformed, leading to the adjustment of model residuals to a normal distribution, which we verified by normal probability plots.

For each of our focal females (*n* = 35 individuals from 15 litters), we calculated, within their litter of origin, the body mass ranking relative to their (male and female) siblings, using the body masses determined on postnatal day 9 (i.e., at the youngest age when it was possible to individually mark the animals with black hair dye, i.e., in a low invasive way). This proportional ranking ranged between 0 (lightest pup) and 1 (heaviest pup) and was independent of differences in litter size, thus could be used as predictors within the same multifactorial model (see also below). First, by a multifactorial LMM (Bates et al., 2015), with litter identity and cage identity as random intercept factors, we tested for associations between the ranked body mass (first predictor), litter size (second predictor), and the increase in body mass from postnatal day 9 to 43.

We ran principal component analyses (PCA) based on the (scaled) three different behavioral variables recorded during open field and novel object tests (see above). This was done separately for the variables recorded on postnatal days 32 (*T*_1_) and 43 (*T*_2_). The aim of this analysis was to reduce these three different variables to a single score, expressing individual differences in exploration tendency. The three behavioral parameters considered, the distance covered in the open field arena, the latency to touch the novel object, and the time spent climbing and exploring, have been shown to be significantly repeatable over time in previous studies on the mound‐building mouse (Duparcq et al., 2019; Jardim et al., 2021) (see details on the repeatability of these parameters in Table A of Suppl. Materials). The two latter variables were log[*x* + 1] transformed prior to analysis, as they showed a strong right‐skewed distribution. Consequently, the resulting PCA scores were well adjusted to a normal distribution, facilitating the use of parametric statistics for further analysis (see below; cf. Jardim et al., 2021). The first axes of both PCAs (at *T*_1_, *T*_2_), which showed highly similar loadings in terms of the direction of how the input variables were associated with it, were averaged for later analyses, hereafter referred to as “exploration tendency.” Before averaging, we verified that this exploration score was significantly repeatable over time (*T*_1_, *T*_2_), by using an LMM‐based intraclass correlation with individual identity as a random factor (R package *rptR*, Stoffel et al., 2017).

We assessed individual‐based associations between the 10 different parameters of oxidative status by a multivariate LMM using the R package *MCMCglmm* (Hadfield, 2010), including litter identity and cage identity as random intercept factors. Pair‐wise correlation coefficients between the different parameters of oxidative status were calculated based on the among‐individual variance matrix provided by this model (Houslay & Wilson, 2017).

Coming back to the main questions of the study, we tested in a first step the effects of the females' original litter size (covariate), their proportional within‐litter body mass ranking on postnatal day 9 (earliest time of individual marking; covariate), and their exploration tendency (PCA score; covariate) on the different parameters of oxidative status (8‐OHdG, PCC, CAT, GPx and SOD in liver and skeletal muscle tissue, respectively) by LMMs (Bates et al., 2015). Litter identity and cage identity were used as random intercept factors. Some of the dependent variables (8‐OHdG, GPx, and SOD) were log[*x*] transformed to adjust model residuals to a normal distribution. The success of these transformations was verified by checking the distribution of model residuals by normal probability plots. We considered all possible two‐way interactions between the three covariates (predictors). There was a statistically significant and negative, although a weak correlation between two of these predictors, the ranked within‐litter body mass and exploration tendency (*marginal R* ^2^ = 0.185, *β* = −0.444 ± 0.141 SE, *p* = 0.004). This is in line with previous studies on animal personality, revealing a certain contribution of early life conditions to the emergence of personality differences (e.g., Rödel & Meyer, 2011), in addition to its genetic basis (van Oers & Mueller, 2010). However, the analysis of variance inflation factors (VIF) for all covariates and 2‐way interactions between them (see Table 2) revealed that VIFs were always lower than 3.3, thus showing no indications of interfering (multi)collinearities in any of our models (Faraway, 2006). VIFs were even lower than 1.6 when considering the final models, after the step‐wise exclusion of statistically non‐significant interaction terms (Engqvist, 2005).

In a second step, we substituted the two predictor variables litter size and the proportional within‐litter ranking on postnatal day 9 by the growth in body mass between postnatal days 9 and 43 (i.e., until around 2 weeks after weaning). This was done as we found, that early growth was negatively associated with litter size and positively with the within‐litter ranking in early body mass relative to littermates (see results for details), thus confirming the findings in various other small mammals (Hudson et al., 2011; Mendl, 1988; Rödel, Prager, et al., 2008). As described above, analyses were done by multifactorial LMMs (with litter and cage identity as random factors), separately for all parameters of oxidative status quantified in the liver and in skeletal muscle tissue. We also tested for the interaction of growth with exploration tendency; the latter was included as the second covariate in these models (see Table B in Suppl. Materials). Again, we did not find any indications of interfering collinearities, as VIFs were always lower than 1.6. We obtained the same results when using the growth from postnatal days 32 to 43 (instead of from postnatal days 9 to 43) as covariate for these analyses (see alternative statistics in Table C in Suppl. Materials).

LMMs were checked for normal distribution of model residuals by normal probability plots, and we verified that variances were homogeneous by plotting residuals versus fitted values (Faraway, 2006). *p*‐values were calculated by corrected *F*‐tests with Satterthwaite approximation (Bolker et al., 2009). Statistically, non‐significant interaction terms were eliminated from the models before these were re‐calculated (Engqvist, 2005). For all statistically significant LMM, we calculated the *marginal R* ^2^ (Nakagawa et al., 2017), which can be interpreted as the proportional variance explained by the fixed effects (R package *partR2,* Stoffel et al., 2021). We controlled for the potential occurrence of false positives in our set of multiple models (*n* = 10 different models, see Table 2) by applying an *α*‐level correction (Benjamini & Hochberg, 1995). All initially statistically significant *p*‐values reported in the table remained below the corrected *α* level (same for Tables A, B in Suppl. Materials). In general, in our results section, statistically significant results (*p* < 0.05) and statistically non‐significant results (*p* > 0.05) are hereafter referred to as “significant” and “non‐significant.”

## 3 RESULTS

### 3.1 Effects of litter size and within‐litter body mass ranking on growth

#### 3.1.1 Litter size effects

The analysis of a larger dataset from our breeding stock (*n* = 87 and 34 litters, respectively) revealed that the mean body mass on postnatal day 1 (LMM: *F*_1,74_ = 13.55, *marginal R* ^2^ = 0.141, *β* = −0.350 ± 0.095 SE, *p* < 0.001) as well as the pre‐weaning growth from postnatal day 1 to day 20 (*F*_1,31_ = 7.91, *marginal R* ^2^ = 0.189, *β* = −0.440 ± 0.156 SE, *p* = 0.008) were significantly and negatively associated with litter size. That is, mound‐building mouse pups from smaller litters were on average heavier shortly after birth and showed a higher pre‐weaning growth.

When only considering growth from postnatal days 32 to 43 (weaning was at day 28), litter size also showed a significant and negative effect indicating a lower growth in juveniles from larger litters during the first few weeks after weaning (*F*_1,32_ = 4.374, *marginal R* ^2^ = 0.083, *β* = −0.293 ± 0.140 SE, *p* = 0.044).

Finally, we also considered in our analysis the increase in body mass between postnatal day 44 until the time of sampling of oxidative status parameters at day 145 (sexual maturity in the mound‐building mouse is at around day 70; Busquet et al., 2009). However, during this period, we did not find a significant association between litter size and growth (*F*_1,14_ = 0.517, *marginal R* ^2^ = 0.014, *β* = 0.133 ± 0.185 SE, *p* = 0.484).

#### 3.1.2 Effects of within‐litter body mass ranking

Based on the data from our 35 focal females, the growth from postnatal days 9 to 43 was significantly and positively associated with the relative, within‐litter body mass ranking on postnatal day 9 (*F*_1,30_ = 13.795, *marginal R* ^2^ = 0.258, *β* = 0.398 ± 0.139 SE, *p* < 0.001). That is, females with a higher‐ranked body mass relative to their littermates showed a higher growth at least until 2 weeks after weaning. Such a significant and positive association was also apparent when only considering early postweaning growth from day 32 to day 43 (*F*_1,32_ = 14.056, *marginal R* ^2^ = 0.259, *β* = 0.525 ± 0.140 SE, *p* < 0.001). However, we did not find a significant effect of within‐litter ranking in starting mass (measured on postnatal day 9) on later growth between postnatal days 44 and 145 (*F*_1,31_ = 0.513, *marginal R* ^2^ = 0.014, *β* = −0.123 ± 0.172 SE, *p* = 0.479).

### 3.2 Consistent individual differences in exploration tendency

We quantified individual differences in exploration tendency of the 35 focal females for which we measured parameters of oxidative status later on. PCAs based on the three behavioral parameters recorded during open field and novel object tests, and separately calculated for *T*_1_ (tests on postnatal day 32) and for *T*_2_ (postnatal day 43), revealed a first axis explaining 48.4% (*T*_1_) and 51.7% (*T*_2_) of the variation of the data (eigenvalues *T*_1_: 1.45; *T*_2_: 1.55), respectively. The eigenvalues of all further axes were <1, and thus these axes were not considered in our analysis. The distance covered in the open field arena (loadings, *T*_1_: +0.594, *T*_2_: +0.276) and the time spent climbing and exploring the novel object (*T*_1_: +0.446, *T*_2_: +0.704) were positively associated, and the latency to touch the novel object (*T*_1_: –0.669, *T*_1_: −0.654) was negatively associated with the score of the first axis. This score, hereafter refer to as “exploration tendency,” was significantly repeatable across the two test sessions (LMM‐based intraclass correlation: *R*_ICC_ = 0.567, *p* < 0.001).

### 3.3 Factors affecting parameters of oxidative status during adulthood

#### 3.3.1 Oxidative DNA damage

**FIGURE 1:**
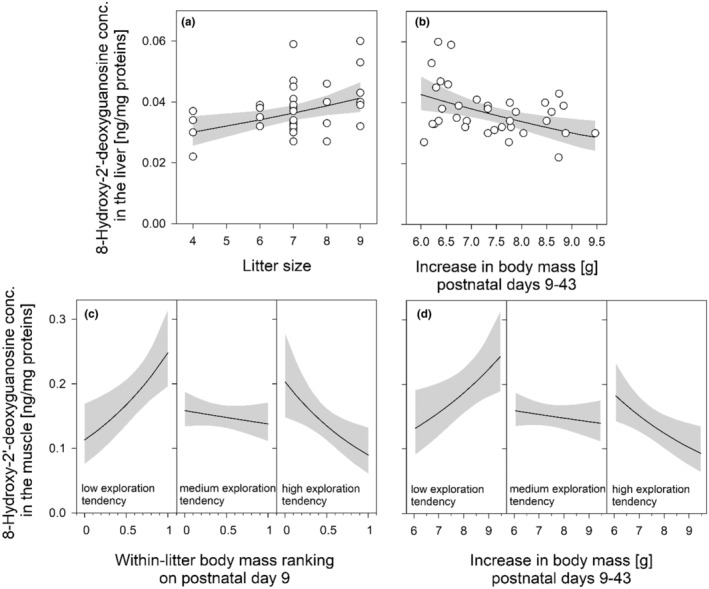
Association between the original litter size of female mound‐building mice (*n* = 35 females from 15 litters) and their 8‐hydroxy‐2′‐deoxyguanosine concentration (as a marker of oxidative DNA damage) in the liver at adult age (4.8 months). The three conditions plotted for each significant interaction are exemplary categorizations (low: 10% percentile, medium: 50% percentile, high: 90% percentile) of the females' within‐litter body mass ranking prior to weaning (continuous variable, see Table 2). The regression line with 95% confidence intervals (gray shading) is based on parameter estimates of a linear mixed‐effects model given in Table 2a.

*Liver tissue*—The 8‐OHdG (8‐hydroxy‐2'‐deoxyguanosine) concentration in the liver was significantly and positively associated with litter size (*marginal R* ^2^ = 0.156, Table 2a), indicating that females born to larger litters had a higher degree of DNA damage in this tissue (Figure 1a).

When substituting litter size by the growth in body mass from postnatal days 9 to 43 (*marginal R* ^2^ = 0.202, *p* = 0.007, details in Table B in Suppl. Materials) or by postweaning growth from days 32 to 43 (*marginal R* ^2^ = 0.157, *p* = 0.017, Table C in Suppl. Materials), these variables also showed significant associations with 8‐OHdG levels in the liver, indicating higher DNA damage in this tissue in individuals with a lower growth (Figure 1b).

*Skeletal muscle tissue*—The concentration of 8‐OHdG in the muscle was significantly explained by the interaction between the females' within‐litter body mass rank and their exploratory tendency (*marginal R* ^2^ = 0.301, Table 2b). This interaction indicates that in females with low exploration tendency, oxidative DNA damage in the muscle was notably higher in individuals with relatively heavy pup body mass compared to lighter littermates, whereas in individuals with high exploration tendency, this positive association tended to inverse (Figure 1c).

Such significant interactions were also apparent when substituting the within‐litter body mass ranking and litter size by the growth in body mass from postnatal days 9–43 (*marginal R* ^2^ = 0.253, *p* = 0.004, Table B in Suppl. Materials), or by the postweaning growth from days 32–43 (*marginal R* ^2^ = 0.293, *p* < 0.001, Table C in Suppl. Materials). That is, in females with low exploration tendency, oxidative DNA damage in the muscle was notably increased with increasing growth, whereas in females with higher exploration tendency, such a positive association was absent and tended to inverse (Figure 1d).

#### 3.3.2 Oxidative protein damage

There were no significant effects by any of the predictors considered on PCC concentration, a marker of oxidative protein damage, either in the liver or in skeletal muscle tissue (all *p* > 0.10; Table 2c,d, and Tables A, B in Suppl. Materials).

#### 3.3.3 Catalase activity

Liver tissue—There were no significant effects of any of the predictors considered on CAT activity in this tissue (all *p* > 0.10; Table 2e, and Tables A, B in Suppl. Materials).

**FIGURE 2:**
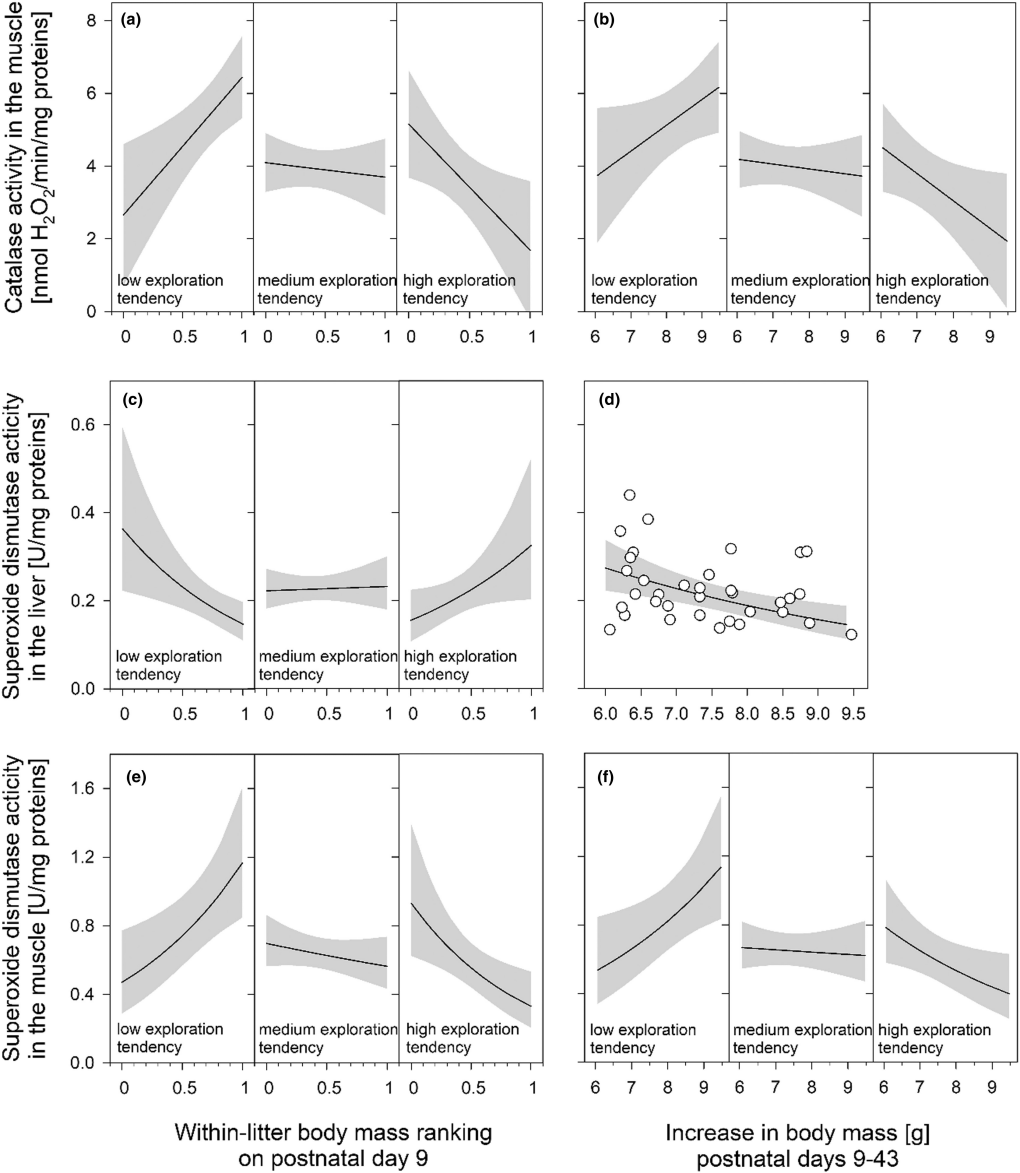
Interactive effects of exploratory activity (PCA score, see text) and the ranked within‐litter pup body mass (0 = lightest, 1 = heaviest pup per litter) on different parameters of oxidative status measured in adult female mound‐building mice (*n* = 35 animals from 15 litters; 4.8 months old). The three conditions plotted for each significant interaction are exemplary categorizations (low: 10% percentile, medium: 50% percentile, high: 90% percentile) of the females' within‐litter body mass ranking prior to weaning (continuous variable, see Table 2). The regression lines with 95% confidence intervals (gray shading) are based on parameter estimates of linear mixed‐effects models given in Table 2.

*Skeletal muscle tissue*—CAT activity in the muscle was significantly explained by the interaction between the animals' within‐litter body mass rank and their exploratory tendency (*marginal R* ^2^ = 0.279, Table 2f). In low‐exploratory females, CAT was notably increased along with increasing within‐litter body mass ranking as a pup. In females with higher exploratory tendency, this positive association between body mass ranking and CAT disappeared and tended to inverse (Figure 2a).

When substituting within‐litter body mass ranking and litter size by the growth from postnatal days 9–43 (*marginal R* ^2^ = 0.224, *p* = 0.021; Table B in Suppl. Materials) or in postweaning growth from days 32 to 43 (*marginal R* ^2^ = 0.296, *p* = 0.001; Table C in Suppl. Materials), there were also significant interactions between growth and exploration tendency. In low‐exploratory females, muscle CAT activity increased with increasing growth. However, in females with higher exploratory tendency, CAT activity generally remained at a lower level, and the positive association with growth turned around (Figure 2b).

#### 3.3.4 Glutathione peroxidase activity

We did not find significant effects by any of the predictors considered, neither on GPx activity in the liver nor in the muscle (all *p* > 0.05, Table 2g,h and Tables A, B in Suppl. Materials).

#### 3.3.5 Superoxide dismutase activity

*Liver tissue*—The significant interaction (Table 2i) revealed that SOD activity in the liver was negatively associated with their within‐litter body mass ranking in less exploratory individuals, but was positively associated with this variable in more exploratory ones (*marginal R* ^2^ = 0.159, Figure 2c).

When substituting the within‐litter body mass ranking and litter size by the growth from postnatal day 9 to day 43 (*marginal R* ^2^ = 0.217, *p* = 0.006; Table B in Suppl. Materials) or by postweaning growth from days 32 to 43 (*marginal R* ^2^ = 0.162, *p* = 0.008; Table C in Suppl. Materials), the latter variables showed significant and negative associations with hepatic SOD activity. That is, the higher the growth was, the lower was the activity of SOD in this tissue (Figure 2d).

*Skeletal muscle tissue*—In the muscle, the significant interaction between the within‐litter body mass ranking on postnatal day 9 and the animals' exploration tendency predicted a contrasting pattern to that of the liver (Table 2j). In low exploratory individuals, the activity of SOD in the muscle increased with the relative pup body mass of the females compared to their littermates, whereas in more exploratory individuals the direction of this association tended to be inversed (*marginal R* ^2^ = 0.261, Figure 2e).

When rerunning the model by substituting body mass ranking and litter size by the growth from postnatal days 9 to 43 (*marginal R* ^2^ = 0.210, *p* = 0.008; Table B in Suppl. Materials) or by postweaning growth from days 32 to 43 (*marginal R* ^2^ = 0.249, *p* = 0.001; Table C in Suppl. Materials), we again found significant interactions between these variables and exploration tendency. These interactions revealed that in low‐exploratory individuals, SOD activity in the muscle increased with increasing growth, whereas this positive association disappeared in individuals with higher exploration tendency (Figure 2f).

## 4 DISCUSSION

### 4.1 Summary of key results

In relation to the main goal of our study, our findings on female mound‐building mice demonstrate long‐term effects of parameters experienced during early life on the oxidative status during adult stage. This was evident by the higher levels of oxidative damage to DNA (i.e., higher 8‐OHdG concentrations) in the liver of animals stemming from larger litters, suggesting long‐lasting consequences of increased sibling competition during early life.

Furthermore, several oxidative status parameters were associated with the animals' relative starting body mass differences within the litter, and these associations were modulated by individual differences in exploration tendency (as evident by the significant interactions between relative starting mass and exploration tendency). First, oxidative DNA damage in skeletal muscle tissue was higher in animals with a relatively higher body mass as pups, but only in less exploratory individuals, that is, in a phenotype typically characterized by a higher adrenocortical activity (Baugh et al., 2017; Carere et al., 2003; Lavergne et al., 2019; Lendvai et al., 2011; Montiglio et al., 2012; Rossi et al., 2018; Stöwe et al., 2010). Furthermore, activity levels of the antioxidants CAT and SOD in skeletal muscle tissue were increased in such animals.

### 4.2 Effects of litter size and within‐litter body mass ranking on growth

Our study confirms a negative association between litter size and early growth, as it has been shown in a wide range of other small mammals (Mendl, 1988; Rödel, Prager, et al., 2008). Furthermore, individuals with a lighter relative starting mass compared to littermates showed a lower early growth. The latter finding is also in accordance with studies in other small altricial mammals, and might be explained by the fact that heavier pups are typically occupying more central, energetically more advantageous positions in the litter huddle, leading to feedback loops positively affecting their growth (Bautista et al., 2010; Rödel, Bautista, et al., 2008).

The lower growth in individuals from larger litters and in such with a relatively lower starting mass was still apparent during the early postweaning period with ad libitum access to food. Thus, given the consistent effects of litter size and relative starting mass before and at least for some time after weaning, we conclude that there were no indications of postweaning compensatory growth (cf. Sikes, 1998) of lighter individuals in the mound‐building mouse.

### 4.3 Litter size effects on parameters of oxidative status

Indications for an increase in oxidative stress in offspring from broods or litters experimentally enlarged in size have already been found in studies on birds (Bourgeon et al., 2011; Gil et al., 2019) and in one on mammals (Gibson et al., 2015). Furthermore, in a study on wild Eurasian kestrels (*Falco tinnunculus*), nestlings from naturally larger brood sizes showed the highest levels of oxidative stress (Costantini et al., 2006). Extending these findings, our study shows for the first time that the natural variation in litter size has the potential to exert such effects in the long‐term, as evident by the increased hepatic 8‐OHdG levels in mature individuals stemming from larger litters. Our study did not reveal compensatory postweaning growth in offspring born to larger litters. Thus, there is no support for the hypothesis that the observed long‐term effects, that is, the higher liver DNA damage in individuals from larger litters, were driven by a higher ROS production related to enhanced, compensatory growth in such individuals (cf. Smith et al., 2016).

One of the possible pathways through which early life conditions, such as growing up in different‐sized litters, may disrupt the oxidative status homeostasis is via increased levels of circulating glucocorticoids, as a consequence of chronic stress (Costantini et al., 2011; Spiers et al., 2015). A higher behavioral activity including increased competition among offspring from larger compared to smaller litters can be observed in some polytocous mammals characterized by scramble competition (e.g., laboratory rat: Bautista et al., 2010) as well as by direct contest for mother's milk (e.g., domestic pig *Sus scrofa:* Kobek‐Kjeldager et al., 2020). The stress‐inducing character of increased sibling competition in larger litters is further supported by some studies showing increased concentrations of circulating glucocorticoids (guinea pigs *Cavia aperea:* Fey & Trillmich, 2008) or a higher adrenocortical capacity in mounting a stress response (Alpine marmots *Marmota marmota:* Cohas et al., 2021) in such offspring before or around weaning. At first sight, it appears unexpected that challenging conditions experienced early in life translate into changes in oxidative status during adulthood, given that oxidative (DNA) damage can be repaired enzymatically, at least to a certain extent (Cooke et al., 2003). Base and nucleotide excision repair mechanisms cleave 8‐OHdG from DNA (Halliwell & Gutteridge, 2015); thus our results might suggest an increased generation of DNA damage, but also increased repair activity. Whatever the exact molecular mechanism, our findings are in line with some other studies, highlighting that early life stress can lead to long‐term alterations of an animal's oxidative phenotype. For example, laboratory mice from experimentally enlarged litters showed consistently decreased aconitase activities around weaning and when reaching maturity (Gibson et al., 2015), indicative of increased oxidative damage in such individuals (Yan et al., 1997). In Japanese quails (*Coturnix japonica*), enduringly stressful conditions experienced during pre‐ and post‐natal development led to long‐term shifts in different antioxidant defenses in the blood and in post‐mitotic neuronal tissue (Marasco et al., 2013).

Interestingly, in the current study, animals with (litter size‐dependent) increased early growth rates also showed an increased activity of the antioxidant SOD in the liver (see Figure 1d), which is contrary to findings obtained in some studies in birds reporting lower antioxidant activities in enlarged broods (Bourgeon et al., 2011; Gil et al., 2019). Furthermore, in our study, higher 8‐OHdG levels were associated with higher activities of the antioxidants SOD and CAT, both in the liver as well as in muscle tissue (see Table 1). In contrast, several studies highlight negative correlations, that is, higher levels of oxidative damage in individuals characterized by a lower antioxidant capacity, in particular in energetically challenged or sick individuals (Kapusta et al., 2018; Tabur et al., 2015). However, as discussed above, challenging early life conditions, such as a more competitive environment in larger litters, may not only induce increased oxidative damage during early postnatal life but also cause longer‐lasting, possibly priming effects (Gibson et al., 2015; Marasco et al., 2013). Thus, we hypothesize that the increased antioxidant enzyme activity observed during adult stage may represent the body's protective response to such long‐term effects on cellular ROS production.

**TABLE 1:** Associations between the different parameters of oxidative stress, based on measurements taken from 35 adult females stemming from 15 litters. Analysis by a multivariate LMM including litter identity and cage identity as random factors. Correlation coefficients are given; statistically significant (*p* < 0.05) negative effects are indicated in red and significant positive effects are indicated in blue

	8‐OHdG liver	8‐OHdG muscle	PCC liver	PCC muscle	CAT liver	CAT muscle	GPx liver	GPx muscle	SOD liver	SOD muscle
8‐OHdG liver		(−0.139)	(−0.071)	(−0.086)	**+0.540**	(−0.135)	**−0.373**	**−0.385**	**+0.712**	(−0.150)
8‐OHdG muscle			(+0.103)	(−0.026)	(−0.024)	**+0.909**	(−0.068)	(+0.046)	(−0.279)	**+0.973**
PCC liver				(−0.302)	(−0.064)	(+0.151)	(−0.001)	(−0.097)	(−0.110)	(0.125)
PCC muscle					(+0.188)	(+0.168)	(−0.174)	(+0.235)	(+0.064)	(−0.043)
CAT liver						(−0.024)	(+0.013)	(−0.084)	**+0.606**	(−0.083)
CAT muscle							(−0.067)	(+0.170)	(−0.354)	**+0.894**
GPx liver								**+0.390**	**−0.374**	(+0.014)
GPx muscle									(−0.341)	(−0.004)
SOD liver										(−0.338)
SOD muscle										

*Note*: Abbreviations: 8‐OHdG, 8‐hydroxy‐2'‐deoxyguanosine (marker of oxidative DNA damage); CAT, Catalase activity (antioxidant); GPx, Glutathione peroxidase activity (antioxidant); PCC, Protein carbonyl content (marker of oxidative protein damage); SOD, Superoxide dismutase activity (antioxidant).

**TABLE 2:** Effects of exploration tendency (PCA score), litter size, and the ranked pup body mass relative to litter siblings on postnatal day 9 (proportional within‐litter score between 0 and 1) on parameters of oxidative damage (a–d) and on the activity of antioxidants (e–j) activity in the liver and in skeletal muscle tissue of adult female mound‐building mice. Data stem from 35 subjects from 15 litters. Sampling of oxidative parameters around postnatal day 145; see short‐cut definition in Table 1. Analysis by multifactorial LMMs including cage identity and litter identity as random factors, with Satterthwaite‘s approximate *F*‐tests. *P*‐values given in bold are still significant after controlling for false discovery rate due to multiple testing (Benjamini & Hochberg, 1995)

Dependent variables	Predictors	*F* (*df*)	*β* ± SE	*p*
(a) 8‐OHdG concentration in the liver	Exploration tendency *E*	0.054 (1,31)	−0.043 ± 0.185	0.817
	Litter size *L*	6.478 (1,31)	0.417 ± 0.164	**0.016**
	Pup body mass rank *R*	0.258 (1,31)	0.093 ± 0.184	0.615
	*E* × *R*	2.985 (1,30)	0.269 ± 0.156	0.094
	*E* × *L*	3.609 (1,29)	0.265 ± 0.139	0.067
	*L* × *R*	0.479 (1,28)	−0.187 ± 0.270	0.494
(b) 8‐OHdG concentration in the muscle	Exploration tendency *E*	0.939 (1,30)	−0.168 ± 0.174	0.340
	Litter size *L*	0.029 (1,17)	0.034 ± 0.197	0.867
	Pup body mass rank *R*	0.259 (1,28)	−0.077 ± 0.152	0.615
	*E* × *R*	10.544 (1,30)	−0.470 ± 0.145	**0.003**
	*E* × *L*	0.005 (1,26)	0.013 ± 0.188	0.946
	*L* × *R*	0.084 (1,22)	−0.044 ± 0.152	0.774
(c) PCC in the liver	Exploration tendency *E*	0.192 (1,28)	−0.090 ± 0.205	0.665
	Litter size *L*	0.024 (1,14)	0.028 ± 0.183	0.879
	Pup body mass rank *R*	0.212 (1,31)	−0.093 ± 0.203	0.649
	*E* × *R*	0.014 (1,27)	−0.022 ± 0.183	0.905
	*E* × *L*	1.102 (1,28)	−0.167 ± 0.159	0.303
	*L* × *R*	0.754 (1,28)	−0.279 ± 0.321	0.393
(d) PCC in the muscle	Exploration tendency *E*	0.324 (1,29)	−0.116 ± 0.204	0.573
	Litter size *L*	0.185 (1,15)	0.080 ± 0.186	0.674
	Pup body mass rank *R*	0.875 (1,30)	0.031 ± 0.199	0.875
	*E* × *R*	2.304 (1,30)	−0.258 ± 0.172	0.143
	*E* × *L*	0.016 (1,28)	−0.029 ± 0.234	0.901
	*L* × *R*	0.234 (1,29)	0.105 ± 0.217	0.632
(e) CAT activity in the liver	Exploration tendency *E*	0.088 (1,31)	−0.059 ± 0.198	0.769
	Litter size *L*	0.221 (1,15)	0.097 ± 0.207	0.645
	Pup body mass rank *R*	0.079 (1,27)	0.051 ± 0.181	0.781
	*E* × *R*	1.975 (1,30)	0.239 ± 0.170	0.170
	*E* × *L*	0.057 (1,27)	−0.054 ± 0.224	0.812
	*L* × *R*	0.225 (1,20)	−0.088 ± 0.186	0.640
(f) CAT activity in the muscle	Exploration tendency *E*	1.350 (1,30)	−0.205 ± 0.177	0.254
	Litter size *L*	0.025 (1,17)	−0.030 ± 0.192	0.876
	Pup body mass rank *R*	0.012 (1,28)	−0.018 ± 0.162	0.193
	*E* × *R*	9.326 (1,30)	−0.439 ± 0.144	**0.005**
	*E* × *L*	1.281 (1,25)	0.134 ± 0.118	0.269
	*L* × *R*	0.001 (1,22)	−0.001 ± 0.241	0.996
(g) GPx activity in the liver	Exploration tendency *E*	0.077 (1,31)	−0.053 ± 0.190	0.784
	Litter size *L*	1.418 (1,16)	0.238 ± 0.200	0.252
	Pup body mass rank *R*	1.998 (1,27)	−0.244 ± 0.173	0.169
	*E* × *R*	2.147 (1,30)	−0.239 ± 0.163	0.153
	*E* × *L*	0.001 (1,28)	0.004 ± 0.211	0.984
	*L* × *R*	1.457 (1,23)	0.216 ± 0.179	0.240
(h) GPx activity in the muscle	Exploration tendency *E*	0.042 (1,30)	−0.042 ± 0.204	0.838
	Litter size *L*	0.228 (1,20)	−0.090 ± 0.188	0.638
	Pup body mass rank *R*	0.568 (1,30)	0.149 ± 0.197	0.457
	*E* × *R*	3.538 (1,29)	0.320 ± 0.170	0.070
	*E* × *L*	0.186 (1,28)	0.098 ± 0.229	0.669
	*L* × *R*	0.209 (1,26)	0.094 ± 0.205	0.651
(i) SOD activity in the liver	Exploration tendency *E*	0.461 (1,31)	−0.121 ± 0.179	0.502
	Litter size *L*	1.444 (1,15)	0.219 ± 0.182	0.248
	Pup body mass rank *R*	0.024 (1,29)	−0.026 ± 0.166	0.878
	*E* × *R*	10.076 (1,30)	0.463 ± 0.146	**0.003**
	*E* × *L*	1.560 (1,26)	0.157 ± 0.125	0.223
	*L* × *R*	0.516 (1,22)	0.177 ± 0.246	0.408
(j) SOD activity in the muscle	Exploration tendency *E*	1.017 (1,30)	−0.171 ± 0.219	0.321
	Litter size *L*	0.002 (1,16)	−0.008 ± 0.169	0.969
	Pup body mass rank *R*	0.712 (1,26)	−0.124 ± 0.198	0.407
	*E* × *R*	10.615 (1,30)	−0.460 ± 0.141	**0.003**
	*E* × *L*	0.063 (1,26)	−0.046 ± 0.182	0.803
	*L* × *R*	0.067 (1,22)	−0.038 ± 0.147	0.798

Nevertheless, this explanation does not exclude the possibility that antioxidant enzymatic activities were decreased when the animals were facing challenging conditions early in life, but may have increased adaptively over lifetime, thus leading to such a positive association during adulthood. Unfortunately, our study based on single endpoint measures only allows restricted insights. Further follow‐up studies using repeated measurements of parameters of oxidative status across different life stages will be useful to explore mechanisms leading to the here observed strong and positive, tissue‐specific associations between parameters of oxidative damage and antioxidant activity (see Table 1).

### 4.4 Effects of relative starting body mass and exploration tendency on oxidative status

Our findings support a link between exploratory tendency and oxidative DNA damage in skeletal muscle tissue. Most other studies exploring personality‐dependent differences in oxidative status report associations with the animals' antioxidant capacity (Costantini et al., 2008, 2012; Herborn et al., 2011; Isaksson et al., 2011; Matsuzawa et al., 2005), and only few have found associations with parameters of oxidative damage. A study on green finches (*Carduelis chloris*), in general accordance with our findings, supports a negative relationship between exploration tendency and oxidative damage, showing that less exploratory individuals had higher concentrations of malondialdehyde, an indicator of cellular lipid peroxidation (Herborn et al., 2011). In the same study, birds with extremely high or low neophobia had lower malondialdehyde concentrations than intermediate responders (Herborn et al., 2011). Furthermore, a study in humans found indications of higher oxidative damage in terms of increased concentrations of malondialdehyde in individuals with high scores of neuroticism (Vida et al., 2018).

However, our study draws a more complex picture, revealing that the increased oxidative DNA damage in muscle tissue of less exploratory phenotypes was only apparent in individuals with a relatively heavier early body mass compared to littermates. First, and as hypothesized upfront, the potentially higher HPA axis activity in low‐exploratory individuals (cf. Baugh et al., 2017; Carere et al., 2003; Lavergne et al., 2019; Lendvai et al., 2011; Montiglio et al., 2012; Rossi et al., 2018; Stöwe et al., 2010), and thus their higher levels of circulating corticosteroids in response to challenge may be a key mechanism explaining the increased oxidative damage in such animals (e.g., Costantini et al., 2012, reviews in: Costantini et al., 2011; Spiers et al., 2015). Second, higher growth rates can carry significant oxidative costs in terms of the accumulation of ROS as a by‐product of an increased metabolism, and a meta‐analysis on this topic suggests that such negative consequences appear particularly visible in terms of increased oxidative damage (review in Smith et al., 2016). This combination of a higher HPA activity and growth rate might explain the increased DNA damage in muscle tissue of faster‐growing individuals with a relatively heavier starting body mass (see Figure 1c,d). Although, as predicted by the significant interaction in our statistical model, only low‐exploratory individuals were affected by such negative consequences, most likely due to their higher sensitivity or predisposition to oxidative stress related to their higher adrenocortical activity (Costantini et al., 2011; Spiers et al., 2015).

Not only the oxidative DNA damage, but also the enzymatic activities of the two antioxidants CAT and SOD in muscle tissue were notably increased in low‐exploratory individuals with a higher relative starting body mass and higher associated growth rate. As already discussed above, this pattern could reflect an adaptive, tissue‐specific upregulation of the antioxidant machinery in animals with particularly high ROS production, thus leading to the positive associations between DNA damage and antioxidant activity levels (see Table 1). However, the single measurements of our study only provide a snap shot of the current condition, and follow‐up studies using repeated measurements may help to better understand cause and consequence of the here observed phenomenon.

In contrast, in the liver, we found indications for a lower SOD activity in such low‐exploratory individuals with a higher relative starting body mass (see Figure 1c). Although little is known about the energetic costs of antioxidant defenses, we speculate that the lower SOD activity in the liver may be the outcome of a trade‐off with observed upregulation in antioxidant capacity in skeletal muscle tissue of such animals. Accordingly, as has been concluded in a study on the oxidative status in common quails (*C. coturnix*), our findings support the assumption that the body's maintenance of muscle integrity might be of higher priority than its protection of the liver during the growing period (Marasco et al., 2021).

Coming back to the effects of litter size on oxidative status; it may appear surprising that the higher early growth rates in offspring from smaller litters did not result in detectable higher oxidative damage in such individuals. One might expect such an effect, in accordance to the above‐discussed negative consequences of higher growth on oxidative DNA damage in initially heavier siblings. One of the reasons for the absence of such an association may be that the effect of litter size on postweaning growth was relatively small (*β* = −0.293 ± 0.140 SE), notably smaller compared to the growth effect driven by within‐litter differences in starting mass (*β* = 0.525 ± 0.140 SE), as evident by the comparison of the absolute values of the standardized regression slopes *β*. Thus, in our correlational study, negative effects of increased sibling competition in larger litters may have simply masked the detectability or may have outweighed the possible detrimental consequences of higher growth in offspring from smaller litters on their oxidative status.

## 5 CONCLUSIONS

Our findings provide some novel insights in the long‐term consequences of early life conditions on oxidative damage and antioxidant activity during adulthood. We show that the naturally occurring variation in litter size is sufficient to exert such effects, in terms of an increased oxidative DNA damage in adult mice born to larger litters. Our findings also suggest synergistic effects of the typically higher stress sensitivity of low‐exploratory phenotypes together with increased oxidative costs in faster growing littermates, possibly leading to higher oxidative DNA damage and to an upregulation of antioxidant defenses in such individuals. Although the character of our study is correlational, and although we can only speculate about the causes and consequences of concomitantly increased levels of oxidative damage and antioxidant levels in some animals, the patterns we observed may help to shed light on potential mechanisms explaining how early life conditions and aspects of animal personality are linked to cellular senescence and other processes of aging (Selman et al., 2012; Yousefzadeh et al., 2021).

## AUTHORS' CONTRIBUTIONS

H.G.R., C.F., R.M., and D.C. conceived and designed the study. H.G.R. supervised the project and statistically analyzed the data. H.G.R. and D.C. wrote the original manuscript. V.J., M.R., L.J., and D.J. carried out experimentation and cared for the animals. V.J. analyzed videos, and V.J., L.J., and D.J. dissected the animals and took tissue samples; D.C. quantified oxidative parameters. All co‐authors contributed to the manuscript by correcting and editing it.

## CONFLICT OF INTEREST

The authors declare that there are no conflicts of interest.

## Supporting information


Appendix S1
