# Supplementary material for: Early life parameters and personality affect oxidative status during adulthood in an altricial rodent
Source: Physiol Rep. 2022 Oct 5;10(19):e15427. doi: 10.14814/phy2.15427 (PMC9535260; doi:10.14814/phy2.15427)
Supplement: Supplementary file 1 — Appendix S1 [file PHY2-10-e15427-s001.docx]

Supplementary Materials

Manuscript submitted to Physiological Reports

Early life parameters and personality affect oxidative status during adulthood in an altricial rodent

Heiko G. Rödel^1*^, Veridiana Jardim^1,2^, Marylin Rangassamy^1^, Ludivine Jaravel^1^, Daphné Jacquet^1^, Raquel Monclús^1^, Christophe Féron^1^, David Costantini^3*^

^a^ *Laboratoire d’Ethologie Expérimentale et Comparée UR 4443 (LEEC), Université Sorbonne Paris Nord, F-93430 Villetaneuse, France*

^b^ *Laboratory of Ethology, Ecology and Evolution of Social Insects, Department of Experimental Psychology, University of Sao Paulo, São Paulo, Brazil*

^c^ *Unité Physiologie Moléculaire et Adaptation (PhyMA), Muséum National d’Histoire Naturelle, CNRS, CP32, F-75005 Paris, France*

*Corresponding authors.

Email addresses: [heiko.rodel@univ-paris13.fr](mailto:heiko.rodel@univ-paris13.fr) (H.G. Rödel); david.costantini@mnhn.fr (D. Costantini)

**SUPPLEMENTS TO METHODS**

**Repeatability of behavioral variables**

Analyses based on the 35 females of the present study revealed that 2 out of the 3 chosen behavioral parameters were significantly repeatable (table A). The third parameter, the latency to approach the novel object, missed the level of significance when only testing 35 females, although was also significantly repeatable when increasing the sample size with data from other females from our data base to a total of *n* = 86. This underlines the usefulness of all three chosen behavioral parameters (as given in Table A) to contribute to the assessment of individual differences in exploration tendency in the mound building mouse.

**Table A** Repeatability of different behavioral variables quantified during repeated open field and novel object tests carried out in female mound building mice. Data from (a) 35 females stemming from 15 litters, and from (b) 86 females from 42 litters (including the 35 females analyzed separately for (a)). Analyses by linear mixed-model based intra-class correlations (1). Statistically significant results are given in bold.

|  | (a) | |  | (b) | |
| --- | --- | --- | --- | --- | --- |
| Behavioral variables | *R*_ICC_ | *P* |  | *R*_ICC_ | *P* |
| Distance covered in Open field | 0.563 | **< 0.001** |  | 0.425 | **< 0.001** |
| Latency to touch Novel object | 0.182 | 0.139 |  | 0.410 | **< 0.001** |
| Time spent climbing on Novel object | 0.576 | **< 0.001** |  | 0.484 | **< 0.001** |

**SUPPLEMENTS TO RESULTS**

**Analysis using early growth as a predictor**

Growth from postnatal days 9 to 43

**Table B** Effects of exploration tendency (PCA score) and **body mass growth from postnatal days 9 to 43** on parameters of oxidative damage (a-d) and on the activity of antioxidants (e-j) activity in the liver and in skeleton muscle tissue of adult female mound-building mice. Data stem from 35 subjects from 15 litters. Sampling of oxidative parameters around postnatal day 145; see short-cut definition in Table 1. Analyses by multifactorial LMMs including cage identity and litter identity as random factors, with Satterthwaite's approximate *F*-tests. *P*-values given in bold are still statistically significant when controlling for false discovery rate due to multiple testing (2).

| Dependent variables | Predictors | *F* (*df*) | *β* ± SE | *P* |
| --- | --- | --- | --- | --- |
| (a) 8-OHdG concentration | Exploration tendency *E* | 2.696 (1,30) | –0.295 ± 0.180 | 0.111 |
| in the liver | Growth d9-d43 *G* | 8.412 (1,30) | –0.552 ± 0.180 | **0.007** |
|  | *E* × *G* | 0.172 (1,31) | 0.061 ± 0.148 | 0.681 |
| (b) 8-OHdG concentration | Exploration tendency *E* | 1.115 (1,31) | –0.187 ± 0.177 | 0.299 |
| in the muscle | Growth d9-d43 *G* | 0.250 (1,29) | –0.082 ± 0.164 | 0.621 |
|  | *E* × *G* | 10.127 (1,25) | –0.384 ± 0.121 | **0.004** |
| (c) Protein carbonyl content | Exploration tendency *E* | 0.193 (1,31) | –0.093 ± 0.211 | 0.663 |
| in the liver | Growth d9-d43 *G* | 0.936 (1,31) | –0.194 ± 0.201 | 0.341 |
|  | *E* × *G* | 0.360 (1,31) | –0.098 ± 0.163 | 0.553 |
| (d) Protein carbonyl content | Exploration tendency *E* | 0.001 (1,28) | –0.001 ± 0.208 | 0.998 |
| in the muscle | Growth d9-d43 *G* | 1.165 (1,28) | –0.214 ± 0.198 | 0.290 |
|  | *E* × *G* | 0.367 (1,31) | –0.089 ± 0.160 | 0.584 |
| (e) Catalase activity | Exploration tendency *E* | 1.102 (1,30) | –0.208 ± 0.198 | 0.165 |
| in the liver | Growth d9-d43 *G* | 0.596 (1,31) | –0.156 ± 0.202 | 0.170 |
|  | *E* × *G* | 0.842 (1,28) | 0.140 ± 0.153 | 0.437 |
| (f) Catalase activity | Exploration tendency *E* | 1.975 (1,31) | –0.264 ± 0.188 | 0.170 |
| in the muscle | Growth d9-d43 *G* | 0.190 (1,29) | –0.056 ± 0.171 | 0.744 |
|  | *E* × *G* | 6.056 (1,25) | –0.315 ± 0.128 | **0.021** |
| (g) Glutathione peroxidase act. | Exploration tendency *E* | 0.136 (1,31) | 0.077 ± 0.208 | 0.715 |
| in the liver | Growth d9-d43 *G* | 0.170 (1,31) | 0.081 ± 0.196 | 0.683 |
|  | *E* × *G* | 1.376 (1,27) | –0.172 ± 0.147 | 0.251 |
| (h) Glutathione peroxidase act. | Exploration tendency *E* | 0.529 (1,29) | 0.038 ± 0.052 | 0.472 |
| in the muscle | Growth d9-d43 *G* | 1.070 (1,29) | 0.051 ± 0.049 | 0.309 |
|  | *E* × *G* | 3.573 (1,31) | –0.075 ± 0.040 | 0.068 |
| (i) Superoxide dismutase act. | Exploration tendency *E* | 1.095 (1,31) | –0.198 ± 0.189 | 0.030 |
| in the liver | Growth d9-d43 *G* | 8.877 (1,29) | –0.514 ± 0.173 | **0.006** |
|  | *E* × *G* | 1.538 (1,24) | 0.161 ± 0.130 | 0.227 |
| (j) Superoxide dismutase act. | Exploration tendency *E* | 0.717 (1,30) | –0.149 ± 0.176 | 0.404 |
| in the muscle | Growth d9-d43 *G* | 0.007 (1,28) | –0.013 ± 0.163 | 0.935 |
|  | *E* × *G* | 8.470 (1,23) | –0.344 ± 0.118 | **0.008** |

Growth from postnatal days 32 to 43

**Table C** Effects of exploration tendency (PCA score) and **pre-weaning body mass growth from postnatal days 32 to 43** on parameters of oxidative damage (a-d) and on the activity of antioxidants (e-j) activity in the liver and in skeleton muscle tissue of adult female mound-building mice. Data stem from 35 subjects from 15 litters. Sampling of oxidative parameters around postnatal day 145; see short-cut definition in Table 1. Analyses by multifactorial LMMs including cage identity and litter identity as random factors, with Satterthwaite's approximate *F*-tests. *P*-values given in bold are still statistically significant when controlling for false discovery rate due to multiple testing (2).

| Dependent variables | Predictors | *F* (*df*) | *β* ± SE | *P* |
| --- | --- | --- | --- | --- |
| (a) 8-OHdG concentration | Exploration tendency *E* | 1.087 (1,30) | –0.179 ± 0.172 | 0.306 |
| in the liver | Growth d32-d43 *G* | 6.386 (1,32) | –0.430 ± 0.170 | **0.017** |
|  | *E* × *G* | 0.170 (1,31) | 0.055 ± 0.135 | 0.680 |
| (b) 8-OHdG concentration | Exploration tendency *E* | 0.245 (1,31) | –0.181 ± 0.164 | 0.624 |
| in the muscle | Growth d32-d43 *G* | 0.028 (1,28) | –0.019 ± 0.138 | 0.893 |
|  | *E* × *G* | 15.756 (1,27) | –0.414 ± 0.104 | **< 0.001** |
| (c) Protein carbonyl content | Exploration tendency *E* | 0.001 (1,31) | –0.001 ± 0.193 | 0.999 |
| in the liver | Growth d32-d43 *G* | 0.816 (1,31) | –0.164 ± 0.181 | 0.373 |
|  | *E* × *G* | 1.871 (1,31) | –0.195 ± 0.143 | 0.181 |
| (d) Protein carbonyl content | Exploration tendency *E* | 0.335 (1,27) | –0.117 ± 0.202 | 0.568 |
| in the muscle | Growth d32-d43 *G* | 0.094 (1,31) | 0.057 ± 0.187 | 0.762 |
|  | *E* × *G* | 0.003 (1,31) | –0.008 ± 0.146 | 0.954 |
| (e) Catalase activity | Exploration tendency *E* | 2.292 (1,30) | –0.292 ± 0.193 | 0.140 |
| in the liver | Growth d32-d43 *G* | 2.058 (1,30) | –0.241 ± 0.168 | 0.162 |
|  | *E* × *G* | 1.419 (1,28) | 0.151 ± 0.127 | 0.243 |
| (f) Catalase activity | Exploration tendency *E* | 0.886 (1,31) | –0.157 ± 0.167 | 0.354 |
| in the muscle | Growth d32-d43 *G* | 0.134 (1,29) | –0.016 ± 0.140 | 0.907 |
|  | *E* × *G* | 12.704 (1,28) | –0.386 ± 0.108 | **0.001** |
| (g) Glutathione peroxidase act. | Exploration tendency *E* | 0.131 (1,30) | 0.072 ± 0.201 | 0.720 |
| in the liver | Growth d32-d43 *G* | 0.519 (1,29) | 0.126 ± 0.175 | 0.477 |
|  | *E* × *G* | 0.830 (1,29) | –0.122 ± 0.134 | 0.370 |
| (h) Glutathione peroxidase act. | Exploration tendency *E* | 0.030 (1,29) | –0.009 ± 0.052 | 0.864 |
| in the muscle | Growth d32-d43 *G* | 0.012 (1,31) | 0.005 ± 0.048 | 0.914 |
|  | *E* × *G* | 0.609 (1,31) | –0.029 ± 0.038 | 0.441 |
| (i) Superoxide dismutase act. | Exploration tendency *E* | 0.001 (1,32) | –0.002 ± 0.170 | 0.990 |
| in the liver | Growth d32-d43 *G* | 8.232 (1,29) | –0.449 ± 0.157 | **0.008** |
|  | *E* × *G* | 1.0748 (1,28) | 0.127 ± 0.122 | 0.309 |
| (j) Superoxide dismutase act. | Exploration tendency *E* | 0.154 (1,31) | –0.064 ± 0.164 | 0.697 |
| in the muscle | Growth d32-d43 *G* | 0.005 (1,28) | –0.010 ± 0.137 | 0.942 |
|  | *E* × *G* | 13.196 (1,27) | –0.376 ± 0.104 | **0.001** |

**REFERENCES**

1. Stoffel MA, Nakagawa S, Schielzeth H. *rptR*: Repeatability estimation and variance decomposition by generalized linear mixed-effects models. *Methods Ecol Evol* 8: 1639-1644 2017.

2. Benjamini Y, Hochberg Y. Controlling the false discovery rate: a practical and powerful approach to multiple testing. *J Roy Stat Soc Ser B (Stat Method)* 57: 289-300, 1995.
